# A Novel Effect of MARCKS Phosphorylation by Activated PKC: The Dephosphorylation of Its Serine 25 in Chick Neuroblasts

**DOI:** 10.1371/journal.pone.0062863

**Published:** 2013-04-25

**Authors:** Andrea Toledo, Flavio R. Zolessi, Cristina Arruti

**Affiliations:** Laboratorio de Cultivo de Tejidos, Sección Biología Celular, Departamento de Biología Celular y Molecular, Facultad de Ciencias, Universidad de la República, Montevideo, Uruguay; University of Iowa, United States of America

## Abstract

MARCKS (Myristoylated Alanine-Rich C Kinase Substrate) is a peripheral membrane protein, especially abundant in the nervous system, and functionally related to actin organization and Ca-calmodulin regulation depending on its phosphorylation by PKC. However, MARCKS is susceptible to be phosphorylated by several different kinases and the possible interactions between these phosphorylations have not been fully studied in intact cells. In differentiating neuroblasts, as well as some neurons, there is at least one cell-type specific phosphorylation site: serine 25 (S25) in the chick. We demonstrate here that S25 is included in a highly conserved protein sequence which is a Cdk phosphorylatable region, located far away from the PKC phosphorylation domain. S25 phosphorylation was inhibited by olomoucine and roscovitine in neuroblasts undergoing various states of cell differentiation *in vitro*. These results, considered in the known context of Cdks activity in neuroblasts, suggest that Cdk5 is the enzyme responsible for this phosphorylation. We find that the phosphorylation by PKC at the effector domain does not occur in the same molecules that are phosphorylated at serine 25. The *in situ* analysis of the subcellular distribution of these two phosphorylated MARCKS variants revealed that they are also segregated in different protein clusters. In addition, we find that a sustained stimulation of PKC by phorbol-12-myristate-13-acetate (PMA) provokes the progressive disappearance of phosphorylation at serine 25. Cells treated with PMA, but in the presence of several Ser/Thr phosphatase (PP1, PP2A and PP2B) inhibitors indicated that this dephosphorylation is achieved via a phosphatase 2A (PP2A) form. These results provide new evidence regarding the existence of a novel consequence of PKC stimulation upon the phosphorylated state of MARCKS in neural cells, and propose a link between PKC and PP2A activity on MARCKS.

## Introduction

MARCKS (Myristoylated Alanine-Rich C Kinase Substrate) is a ubiquitous natively unfolded protein originally discovered as a major protein kinase C (PKC) substrate in the nervous system [1]. Since then most of its characterization was done taking into account this feature, reinforced by the fact that the majority of its molecular interactions have been found to be concentrated at a very small portion of the protein, the “effector domain” (ED), where all PKC sites are located [2], [3]. This ED, spanning 25 aminoacids, represents less than 10% of the total protein sequence, but it mediates MARCKS electrostatic interaction with membrane phospholipids, actin binding and cross-linking as well as calcium-calmodulin binding. In addition, the amino-terminus is co-translationally myristoylated, and this modification aids in plasma membrane association [4], [5]. Genetic manipulations in mice have been extremely useful in beginning to understand MARCKS functions during development. The inactivation of the Marcks gene causes defects in neural development, such as exencephaly, agenesis of interhemispheric commisures, neuronal ectopia, abnormal retinal lamination and retinal folding [6]. Most of these phenotypic features are rescued by transgenic expression in KO mice of the intact molecule or a non-myristoylatable form [7]. However, a protein form that is nonmyristoylatable and pseudo-phosphorylated at the ED (thus unable to bind to the membrane) was not able to rescue the retinal phenotype, suggesting special functions of the protein in this organ [8]. Interestingly, all defects could be restored by the expression of a mutated form of MARCKS in which the ED was rendered non-phosphorylatable [9], suggesting that phosphorylation by PKC cannot account for all MARCKS functions.

Sometime after the discovery of MARCKS and the ED phosphorylation, other six serine/threonine phosphorylated residues were found in MARCKS purified from membrane fractions of bovine brain, although its cell type origin was not determined [10]. In addition, MARCKS purified from rat brain was found to be a substrate *in vitro* for proline-directed kinases, although the phosphorylated residues were not identified [11]. One of these sites was later recognized as serine 113, which matches the MAPK phosphorylation motif, and was in fact phosphorylated in vitro by this kinase [12]. It is interesting to point out that another proline-directed kinase, the cyclin-dependent Cdk2, phosphorylated recombinant human MARCKS *in vitro* at serine 26 [13]. This is a singular site since it is only found phosphorylated in post-mitotic developing neuronal cells, as we showed some time ago [14], [15]. This serine is located far away from the ED, residing at the amino terminal region of the protein at position 25 in the chick and 26 in several mammalian species (this MARCKS isoform is named S25p-MARCKS while pED-MARCKS designates the form phosphorylated at the ED). The stability of MARCKS S25 phosphorylation depends on the integrity of the actin cytoskeleton [16], perhaps via an intra-chain long range effect originated at the actin-binding site located at the ED.

On one hand, MARCKS has been studied in many different cell types and has been implicated in cross talks between some signal transduction pathways. On the other hand, there is a substantial amount of information concerning its role in secretory and migration activities involving components of the cell cortex (reviewed in [5]). However, information is lacking regarding the putative kinases, other than PKC, acting in developing neural cells as well as about the possible relationships between these multiple phosphorylation sites in this natively unfolded protein. These questions prompted us to perform a search on the origin of phosphorylation at serine 25 as well as on its possible modulation by the phosphorylation at the PKC sites. Consequently, we explored the presence of both phosphorylated ED and S25 at representative stages of neural retina development by Western-blot and *in situ* localization. The finding of some sequence singularities in the protein region where S25 lies, as well as the results from pharmacological assays, indicate that a Cdk is the kinase involved in the phosphorylation of S25. Using phorbol 12-myristate13-acetate (PMA) as a PKC stimulator we were also able to establish how, in these conditions, there is a disappearance of the phosphorylation at S25 and to localize differently phosphorylated MARCKS protein clusters in isolated cells. We also found that MARCK S25 dephosphorylation depends on the activity of a phosphatase inhibitable by calyculin A, but not by tautomycetin or other classical protein phosphatase inhibitors.

As far as we know, this is the first report showing that two different events of MARCKS phosphorylation, that could potentially occur in the same polypeptide chain are mutually exclusive, and that the two resulting MARCKS variants reside in different protein clusters in retinal neuroblasts.

## Materials and Methods

### Animals, Cell Cultures and Pharmacological Treatments

Fertilized hen eggs were kindly supplied by Prodhin Uruguay, and incubated in our laboratory at 37°C in a humidified atmosphere. For all procedures chick embryos were rapidly decapitated once extracted from the shell. E4 and E8 chick embryo neural retinas were excised and the cells isolated and cultured as described [14] using different amounts of dissociated cells at seeding in order to obtain high or low glial cell numbers in the neuroblast cultures. For the kinase inhibition experiments cell suspensions were cultured in DMEM with 5% (E8 cells) or 10% (E4 cells) FCS, at 37°C in a humidified atmosphere containing 5% CO_2_. Cells were incubated with roscovitine, olomoucine or LiCl (Sigma Aldrich, MO, USA) for variable times, as indicated in each figure. For control experiments, cells were incubated with vehicle (DMSO) or NaCl (control for LiCl). For PKC stimulation experiments, E8 neural retina cell suspensions were cultured three days in DMEM with 10% FCS, and then treated with PMA (Sigma Aldrich, MO, USA) or DMSO at the concentrations, and for the times, indicated in each figure. For phosphatase inhibitor treatments, cells were first washed in DMEM without FCS and then incubated in this medium containing okadaic acid (EMD Millipore, MA,USA), cyclosporin A (Fluka, Sigma Aldrich, MO, USA), calyculin A (Cell Signaling, MA,USA ), FK506 (Cell Signaling, MA,USA ), or tautomycetin (Tocris, Bristol,UK/R&D Systems,MN,USA) at different concentrations and for variable times, as indicated in each figure and in the text. For control experiments, cells were incubated with vehicle (DMSO) at the appropriate concentration. For PKC stimulation, PMA was added to the cell cultures without removing the media containing the phosphatase inhibitors or vehicle. After incubation, isolated cells were lysed in electrophoresis buffer, containing SDS and β-mercaptoethanol. For microscopic examination, cells were plated on glass coverslips pre-coated with poly-L-ornithine or laminin (Sigma Aldrich, MO, USA). After incubation they were fixed in 3.7% paraformaldehyde for 30 minutes. For treatments of E8 intact retinas with PMA, retinas were isolated and incubated in DMEM without FCS in the presence of PMA as indicated in the figure. After incubation, retinas were lysed in electrophoresis buffer, or fixed in 3.7% paraformaldehyde for 30 minutes and processed for cryosection as previously described [14].

### Antibodies, Western-blotting and Immunofluorescence, Flotation Assays

Primary antibodies used were mAb 3C3 (mouse monoclonal anti-S25p-MARCKS [17]); MCt (rabbit polyclonal anti-carboxy-terminal MARCKS, kind gift of P. Caroni [18]); Polo52 (polyclonal anti-MARCKS antibody; see production details below); anti-pED-MARCKS (rabbit polyclonal anti-phosphoserine^152/156^ from Sigma Aldrich, MO, USA); Martin D202-D87 (rabbit polyclonal anti-chick MARCKS, kind gift of P. Blackshear [19], [20]), anti-Cdk5 C8 (rabbit polyclonal from Santa Cruz Biotechnology, CA, USA); anti-GSK3β 1H8 (mouse monoclonal, from Virogen, MA, USA); and anti-phospho-GSK3β Ser9 (rabbit polyclonal from Cell Signaling, MA, USA). Nuclear staining was made with Hoechst 33342 (Invitrogen, CA, USA) or propidium iodide (Sigma-Aldrich, MO, USA).

To generate the Polo52 rabbit polyclonal antibody, whole recombinant, His-tagged, chicken MARCKS protein was produced in bacteria. Briefly, MARCKS full coding sequence (from pBC12/pCMV-60k plasmid [19]) was PCR sub-cloned into pET 28 b expression vector and used to transform HMS 174 *E. coli* bacteria. After growth, induction and lysis by standard methods, 1.5–2 mg recombinant MARCKS were affinity purified using a HisTrap HP 5 mL column (GE Healthcare, USA). This was used to produce a rabbit anti-serum according to standard immunization protocols. Briefly, one rabbit was primed by multiple intradermal injections with a total of 500 µg of MARCKS protein in Freund’s adjuvant, followed by two 300 µg boosters by intramuscular injection at days 30 and 54. Bleeding to obtain the antiserum was at day 65. Recombinant protein was produced at the Recombinant Proteins Unit, Institut Pasteur de Montevideo, Uruguay (https://www.pasteur.edu.uy/upr) and the antibody at the Biotechnology Service, Polo Tecnológico de Pando, Universidad de la República, Uruguay (http://www.polotecnologico.fq.edu.uy/). Western-blot characterization of this antibody is shown in Figure S1.

Western-blots were made as previously described [16]. For quantification of protein phosphorylation, sequential immunodetection of phospho-specific and anti-total protein antibodies on the same membrane was performed as described [14]. Densitometry analyses were done using ImageJ 1.43 g software (NIH, MD, USA). For detailed comparative analyses of MARCKS isoforms, the radiographic films were digitalized and false colored using ImageJ. Images were then overlapped to analyze the relative migration of different phosphorylation variants and total MARCKS. Cultured cells were processed for immunofluorescence as described [14], using secondary antibodies coupled to different fluorochromes. Immunolabeled cells were observed and photographed using a Nikon Microphot FXA microscope or a spectral laser scanning confocal microscope, Leica TCS SP5. For each experiment, different images were acquired using the same settings (ie. laser power, gain and offset). Confocal images show either single optical sections or maximum intensity projections, as indicated in each figure. For image acquisition and initial observation, Leica LASAF software was used, while the Fiji version of ImageJ was used for all image processing and generation of videos [21]. Flotation assays were performed according to [22].

### Statistical Analysis

Statistical significance for the experiments using kinase inhibitors was determined using the nonparametric tests Kruskal-Wallis and Wilcoxon-Man Whitney. The values, represented as mean±SEM, were considered to be significantly different when p≤0.05.

## Results

### MARCKS Phosphorylation at the ED and S25 during Normal Retina Development

**Figure 1:**
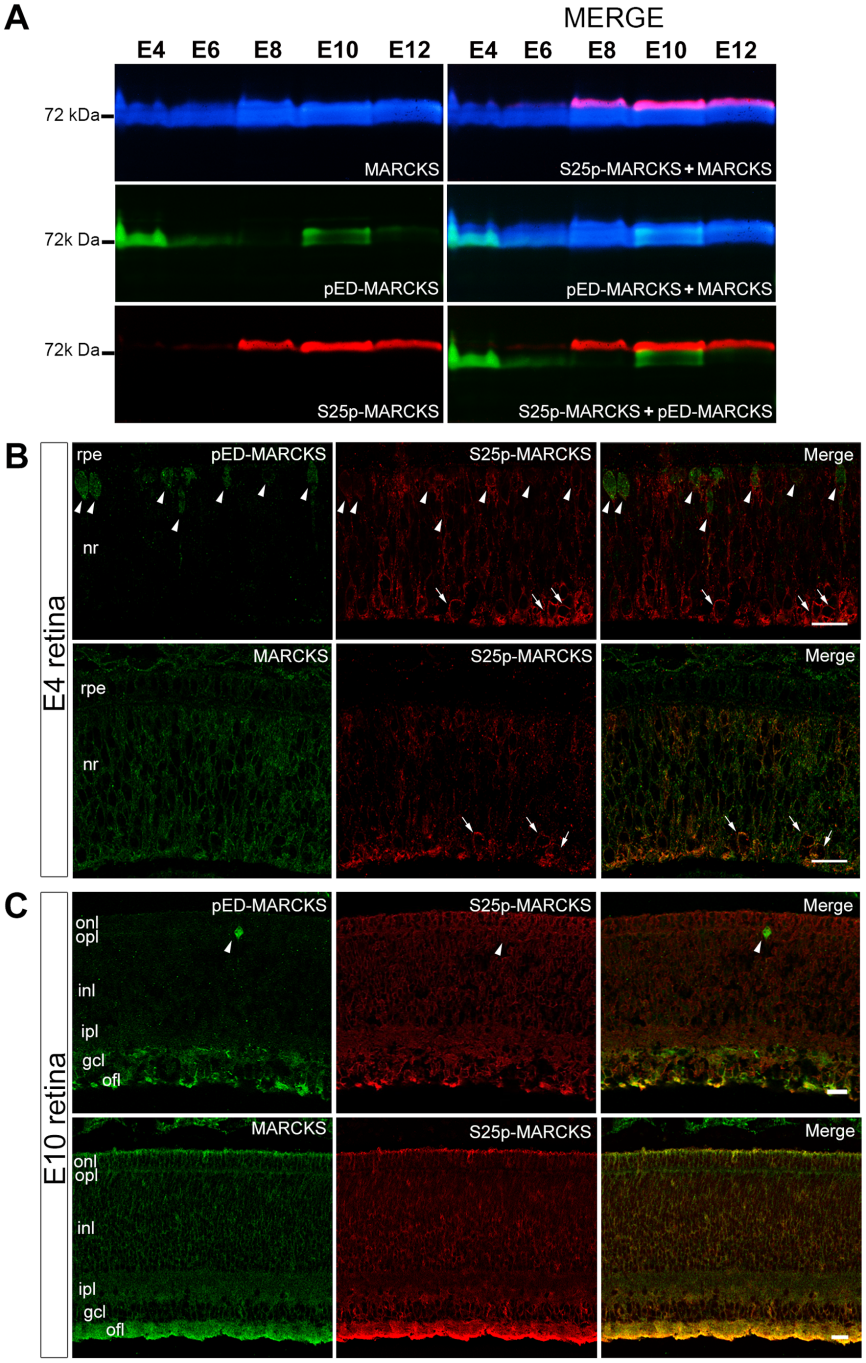
MARCKS phosphorylation at the ED and S25 during chick retina development. (**A**) Sequential Western-blot immunodetections for MARCKS, pED-MARCKS and S25p-MARCKS of E4–E12 chick embryo retinas, as indicated on top of the figure. The immunodetection of each protein isoform is shown in a different false color, and a digital merge is shown on the right. (**B–C**) Single confocal sections of MARCKS, pED-MARCKS and S25p-MARCKS immunodetection on E4 and E10 chick retina cryosections. Arrowheads, mitotic cells at the apical border of the neuroepithelium; arrows, newly differentiating ganglion cells at the basal border. nr, neural retina; rpe, retinal pigment epithelium; gcl, ganglion cell layer; inl, inner nuclear layer; ipl, inner plexiform layer; onl, outer nuclear layer; ofl, optic fiber layer; opl, outer plexiform layer. Scale bars: 20 µm.

To assess the state of MARCKS and its phosphorylation at either ED or S25, soluble fractions of neural retinawere analyzed by Western-blot. As it is shown in Figure 1A, MARCKS migrates as a broad and irregular band at approximately 72 kDa as it is known, given its peculiar aminoacid sequence (its actual MW is around 27.8 kDa [17]). In fact, MARCKS has a highly acidic isoelectric point, impairing SDS binding [2]. Observe that the electrophoresis band has a clearly heterogeneous density, evidencing the known existence of different isoelectric variants [14], [15]. The relative amounts of pED-MARCKS and S25p-MARCKS in the neural retina were found to be different at the developmental stages assayed (Fig. 1A). S25p-MARCKS gradually increased with developmental stage, while the changes in pED-MARCKS immunoreactivity appeared more random, possibly reflecting some particular functional states of the neural retina cells at the moment of isolation. A most interesting fact concerning these phosphorylated polypeptides was their different relative mobility evidenced in the superposed pseudo-colored immunoblots (Fig. 1A). The absence of immunoreactivity overlapping, consistently observed in all the immunoblots made in our experiments, clearly indicated that these phosphorylations do not occur in the same polypeptide chains.

pED-MARCKS was abundantly found in mitotic cells at E4, a stage when many neuroepithelial cells are dividing. Here, S25p-MARCKS was heavily immunodetected on differentiating retinal ganglion cells, while the signal was lower on cells located at the central region of the neural retina wall (Fig. 1B). It is necessary to remark that mAb 3C3 also recognizes a yet unidentified antigen present in mitotic cells (Fig. 1B). At more advanced development stages, as E10, pED-MARCKS was mainly confined to the inner-most retinal layers (optic fiber, ganglion cell and the vitreal portion of the inner plexiform layers). At the same time S25p-MARCKS was found in all retinal layers (Fig. 1C).

### Identification of the Neuroblast-specific Phosphorylation Site S25 in a MARCKS Domain Highly Conserved in Vertebrate Evolution

**Figure 2:**
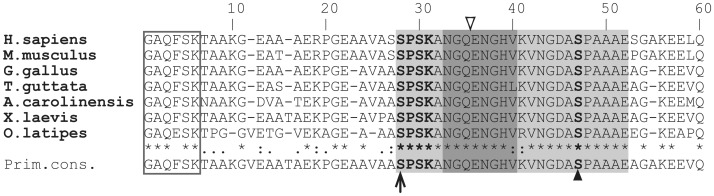
Positioning of S25 at a highly conserved MARCKS region, the MH2. Alignment of the amino-terminus sequences of MARCKS from seven different vertebrate species: two mammals (*H. sapiens* and *M. musculus*), two birds (the chick, *G. gallus* and the zebrafinch, *T. guttata*), one reptile (the lizard *A. carolinensis*), one amphibian (*X. laevis*) and one bony fish (medaka, *O. latipes*). Two highly conserved regions are evident in this portion of the protein: the first six-aminoacid stretch (empty box; excluding initial M), which is the myristoylation domain; and the region that starts with S25 (arrow) and ends with E49, or “MARCKS Homology 2″ (MH2, similar to the one previously reported in [35]; light gray box). The “SPSK” Cdk phosphorylation consensus is highlighted in bold font. In bold font is also highlighted a second serine residue followed by proline, which is also extremely conserved (S44 in the chick; arrowhead). The dark grey box highlights the region originally recognized as conserved in five MARCKS sequences, which is encoded by the region of mRNA splicing [3]). Open arrowhead marks the splicing point.

Nucleotide sequence information about MARCKS is available for five classes of vertebrates: mammals, birds, reptiles, amphibians and bony fishes. Therefore, it is possible to evaluate a representative number of primary structures. Indeed, a comparison of many of them (Fig. 2 and data not shown) confirms early observations on the existence of three main conserved regions: the N-terminal myristoylation consensus, “MH1”; a close-by “MH2”domain, of unknown function, and the effector domain “ED” (see reviews [3], [23]). Figure 2 shows a comparative alignment of the N-terminal peptide sequence of seven putative MARCKS orthologues from five classes of vertebrates. Among these seven species, overall identity is very low, around 29% (not shown). However, identity at the MH2 is 23/25 residues (human S26-E50; Figure 2) whereas at the ED it is 24/25 residues (human K151–K175; not shown). In MH2, the only differences are on V38, substituted by leucine in the zebrafinch (*T. guttata*) and K39, substituted by arginine in medaka fish (*O. latipes*). S25, a site specifically phosphorylated in chick neuroblasts [17], is located at the N-terminus of the MH2 domain (Fig. 2). Interestingly, in all proteins, the residue corresponding to S25 in the chick (human S26) is followed by the stretch “PSK”. This tetrapeptide exactly matches one of the target consensus sequences proposed for cyclin-dependent kinases (Cdks): S/T-P-X-K/R [24]. Therefore, we next tested the hypothesis that a Cdk is MARCKS S25 kinase in chick retina neuroblasts.

### Inhibition of MARCKS Phosphorylation at S25

Cell biology findings showed that MARCKS phosphorylation at S25 disappears in dissociated cells, but that it recovers as a function of time in culture, reaching a steady state one day after plating [16]. As a first approach we took advantage of these data to design inhibition assays for different kinases. We used easily reversible pharmacological kinase inhibition or activation to get a better understanding of the participation of these enzymes in MARCKS phosphorylation, occurring at different cell differentiation and functional states.

**Figure 3:**
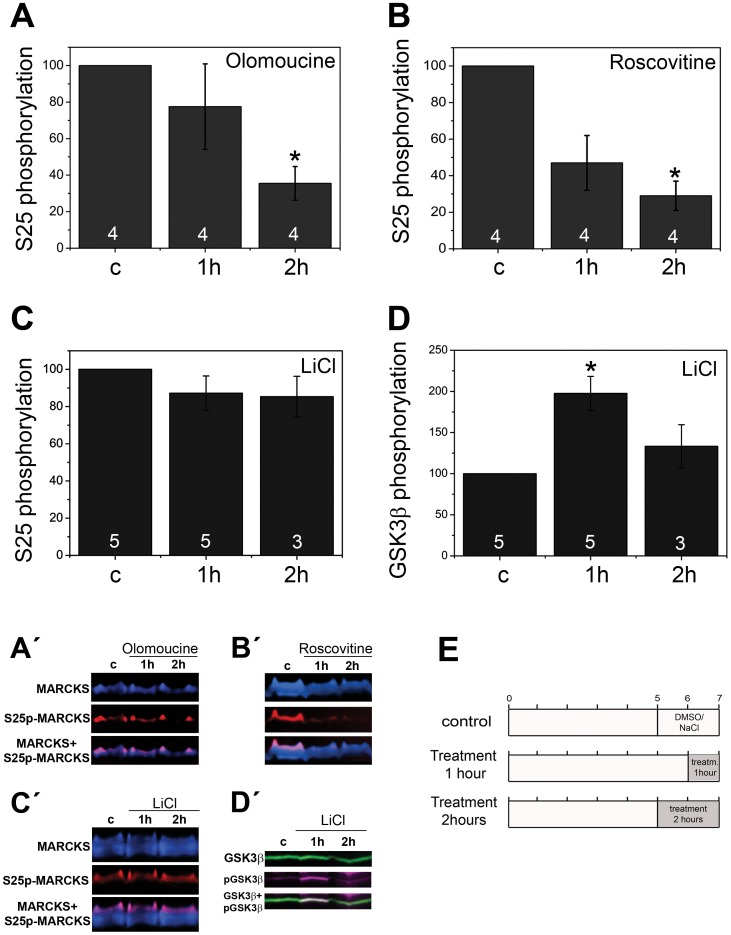
Cdk and GSK3β inhibitors and MARCKS phosphorylation at S25 in cultured retina neuroblasts. (**A–D**) Bar diagrams representing the relative phosphorylation of MARCKS at S25, or GSK3β at S9, as percent of control, after treatment with Cdk and GSK3β inhibitors. Chick E8 retina cells were cultured for 7 hours and treated during the final hour or two with 100 µM olomoucine (A), 25 µM roscovitine (B), or 20 mM LiCl (C and D). Values in C and D were taken from the same experiments. Control cultures were treated with 0.05% DMSO (A and B) or 20 mM NaCl (C and D) for 1 or 2 hours. The values are represented as mean±SEM, *p≤0.05. Statistical significance was determined using the nonparametric tests Kruskal-Wallis and Wilcoxon-Man Whitney. Numbers inside the bars show the “n” for each case (independent experiments). Representative Western-blots are shown in A′–D′, sequential immunodetections are shown in different pseudo-colors. (**E**) Scheme showing the time-course of treatments.

**Figure 4:**
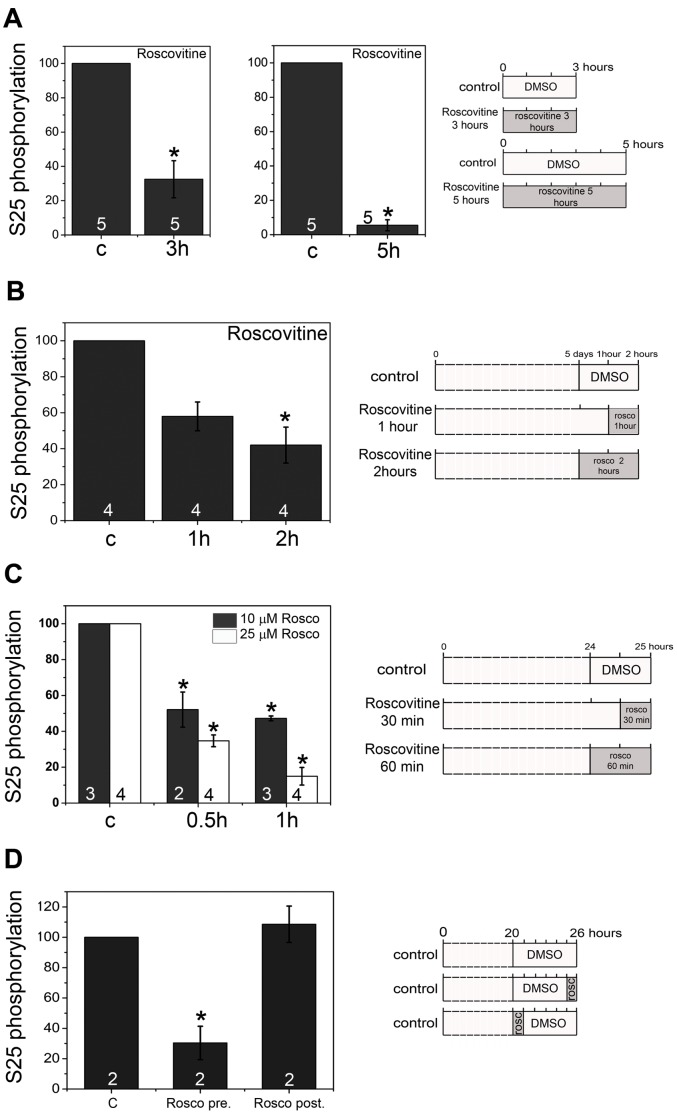
Roscovitine inhibition of MARCKS phosphorylation at S25 in different functional states of retinal cells. E4.5 and E8 retinal cells were treated with the Cdk inhibitor roscovitine after different cell culture protocols, and the relative phosphorylation of MARCKS at S25 assayed by Western-blot. Control cultures were treated with 0.05% DMSO. Mean±SEM, *p≤0.05. Numbers inside the bars show the “n” for each case (independent experiments). Statistical significance was determined using the nonparametric tests Kruskal-Wallis and Wilcoxon-Man Whitney. (**A**) E8 chick retina cells were cultured for 3 or 5 hours and treated during this period with 25 µM roscovitine. (**B**) E8 chick retina cells were cultured for 5 days and treated with 25 µM roscovitine for 1 or 2 hours. (**C**) Peripheral E4.5 chick retina cells cultured during 24 hours were treated during 30 minutes or 1 hour with 10 or 25 µM roscovitine. (**D**) Reversibility of the decrease in relative MARCKS phosphorylation at S25 after roscovitine treatment. Peripheral E4.5 chick retina cells cultured during 20 hours were treated as follows. “Control”: 6 hours DMSO, “Rosco pre”: 1 hour 25 µM roscovitine, then 5 hours DMSO “Rosco post”: 5 hours DMSO, then 1 hour 25 µM roscovitine.

Dissociated retinal cells from E8 embryos were cultured for seven hours *in vitro* and treated with different kinase inhibitors during the last one or two hours, a time in which *de novo* MARCKS phosphorylation should be progressively increasing (Fig. 3E). Two Cdk inhibitors, olomoucine and roscovitine [25], significantly lowered S25p-MARCKS immunoreactivity relative to total MARCKS, as shown in Western-blots from these cells (Fig. 3A, A′, B and B′). However, the inhibition of another kinase, GSK3β, caused no decrease in serine 25 phosphorylation (Fig. 3C, C′, D and D′). In all treatments considered, cell death was not significantly different to controls (not shown). To assess the effects of Cdk inactivation at the onset of the re-phosphorylation period, roscovitine was added before cell attachment and spreading (at the time of cell plating). In this condition there was again an important inhibition of S25p-MARCKS recovery (Fig. 4A). Roscovitine also lowered S25p-MARCKS in cells grown *in vitro* up to a stage of advanced morphological differentiation (Fig. 4B). To determine whether this inhibitor was able to impair MARCKS phosphorylation at the beginning of cell differentiation, we performed experiments on differentiating retinal neuroepithelial cells (see Fig. S2). Incubation of these cells with roscovitine provoked a noticeable reduction of MARCKS phosphorylation at S25, even when a low concentration (10 µM) and short time (30–60 min) exposures were assayed (Fig. 4C). Furthermore, the effect was quickly reversed sometime after roscovitine was withdrawn from the culture medium (Fig. 4D). We confirmed that the treatment was not causing cell death by quantitating the proportion of pycnotic nuclei and cells showing surface blebbing (Table S1). We conclude that a Cdk is responsible for MARCKS S25 phosphorylation at different neuroblast growth and differentiation stages. As it is known that Cdks other than Cdk5 are not active in these post-mitotic cells [26], [27], we subsequently focused on the subcellular localization of Cdk5, and its possible interactions with MARCKS in differentiating neurons.

### Localization of S25p-MARCKS and Cdk5 in Differentiating Neurons

**Figure 5:**
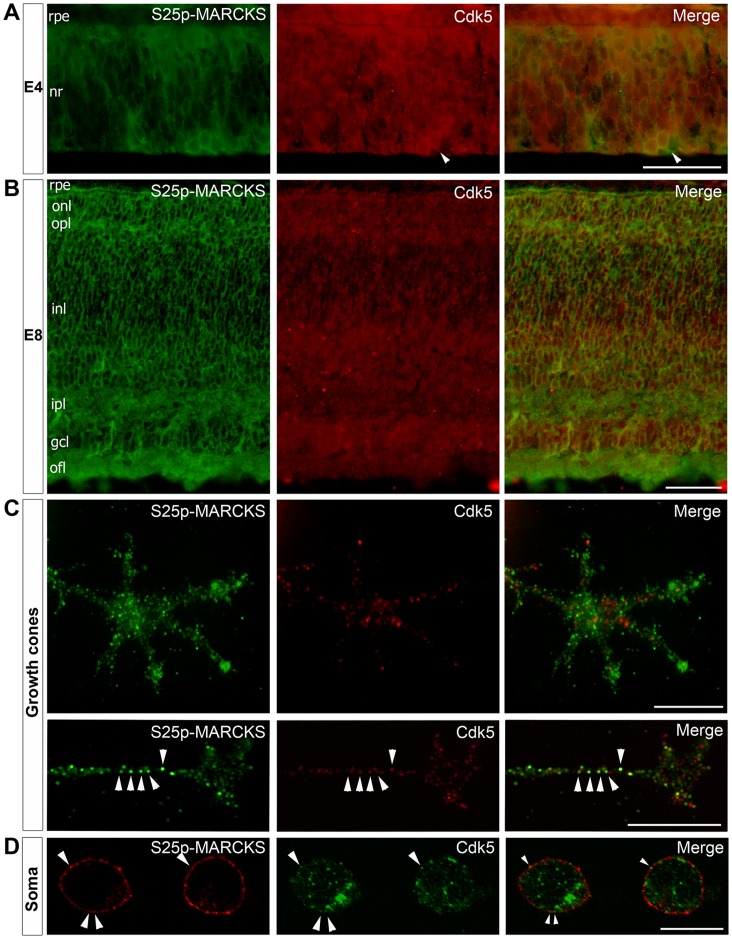
Subcellular distribution of S25p-MARCKS and Cdk5 in chick retinal cells. (**A–B**) Double immunofluorescence detection of S25p-MARCKS and Cdk5 on E4 and E8 chick embryo retina cryosections. Arrowheads indicate newly differentiated neurons, highly enriched in both proteins. gcl, retinal ganglion cells; inl, inner nuclear layer; ipl, inner plexiform layer; nr, neural retina; onl, outer nuclear layer; ofl, optic fiber layer; opl, outer plexiform layer; rpe, retinal pigment epithelium (epifluorescence). Scale bars: 50 µm. (**C–D**) Double immunofluorescence detection of S25p-MARCKS and Cdk5 on neurons cultured from E8 retina. Images in C show growth cones (epifluorescence) while D shows single confocal sections of cell somas. Arrowheads indicate co-localizing clusters. Scale bars: 10 µm.

We previously showed that the first cells containing high amounts of S25p-MARCKS were those placed at the innermost region of the neural retina and that expressed an early neuronal differentiation marker [14]. Double immunostaining of S25p-MARCKS and Cdk5 on E4 chick embryo sections showed that the kinase, even if present in all retinal cells, was more intensely detected in differentiating neurons (Fig. 5A). At more advanced stages (E8) all the retinal layers contained both proteins (Fig. 5B).

Both total MARCKS and S25p-MARCKS were found accumulated as irregular speckles at the cell periphery of cultured differentiating neurons, either at the cell soma, neurites or growth cones [16]. Cdk5 had this same cell distribution but it was mostly not co-localizing with S25p-MARCKS at the level of the protein clusters (Fig. 5C). It was observed that the distribution of both proteins seemed to be different in small and large growth cones. Large growth cones exhibited a complementary distribution of both protein clusters at the region of the growth cone central domain, while they were more intermingled at the peripheral domain of the smaller growth cones. Only some very scarce clusters in the thinnest neurites seemed to contain S25p-MARCKS and Cdk5 (Fig. 5C). Optical sections at the middle regions of the neuroblast soma were very useful to analyze the distribution of both proteins, as they showed a clear peripheral S25p-MARCKS localization while Cdk5 occupied the central cell volume; only some extremely rare Cdk5 clusters appeared at the peripheral regions, very little co-localizing with S25p-MARCKS (Fig. 5D). Although the speckles on cells are probably formaldehyde-fixing artifacts, they may also represent lipid raft-association in living cells [22]. After a separation of cold Triton X-100 extracted cell membranes in a sucrose gradient, we found a small amount of both S25p-MARCKS and Cdk5 present in the low-density fraction, corresponding to detergent-resistant membranes (“lipid rafts”; Fig. S3). Altogether, these results indicate that a small fraction of Cdk5 and S25p-MARCKS may overlap in subcellular compartments associated to the plasma membrane.

### Relationship between the Phosphorylations at the ED and S25 when the Cells are Stimulated by PMA

**Figure 6:**
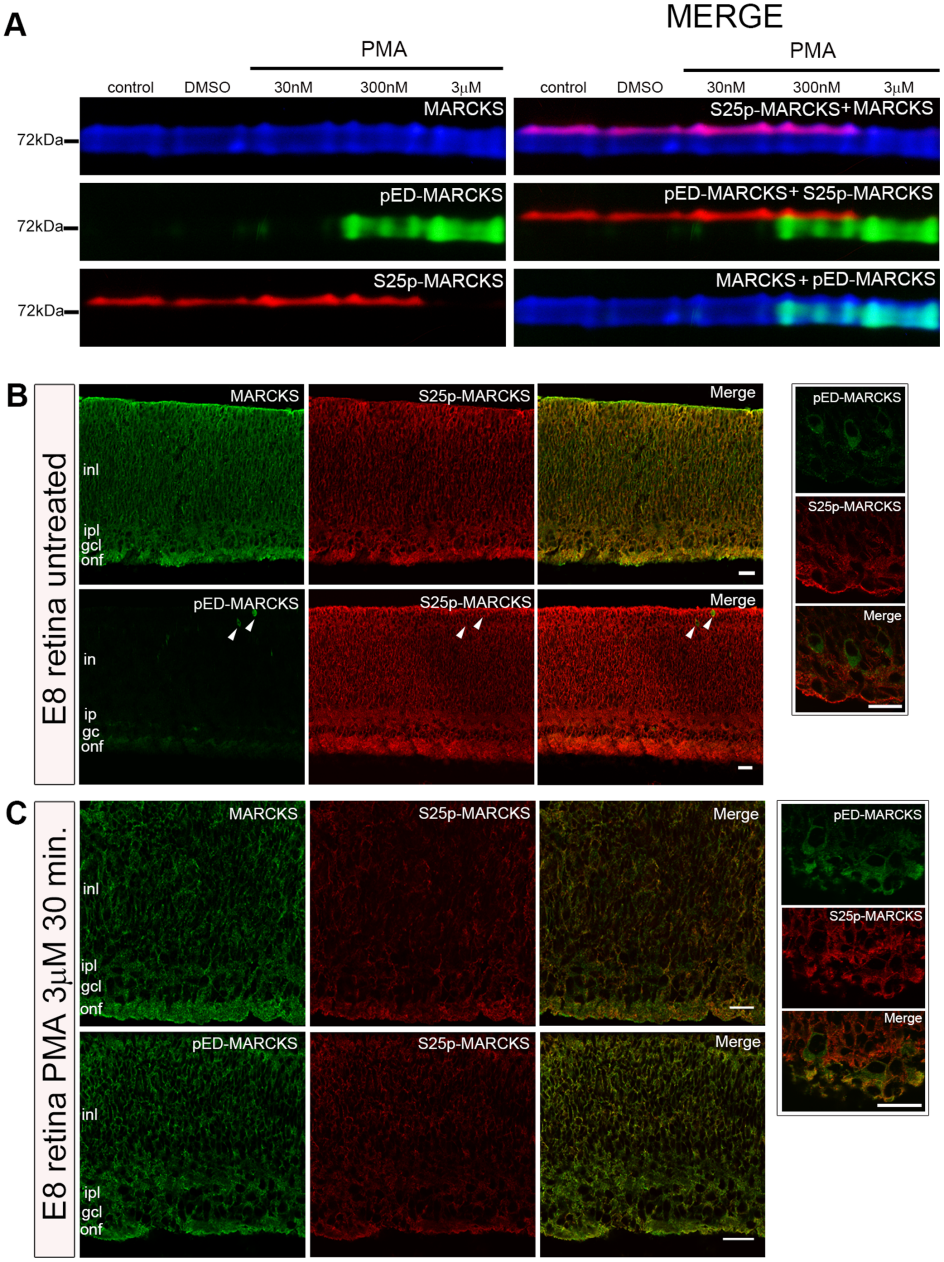
MARCKS phosphorylation at the ED and S25 in E8 chick embryo retinas stimulated with PMA. (**A**) Sequential Western-blot immunodetections for MARCKS, pED-MARCKS and S25p-MARCKS of E8 chick embryo retinas treated with different concentrations of PMA for 30 minutes. There are two controls: untreated and 0,1% DMSO. (**B–C**) Single confocal sections of chick retina cryosections, immunolabeled for MARCKS, pED-MARCKS and S25p-MARCKS. Insets on the right show a higher magnification untreated retinas, at the vitreal margin to highlight the localization of pED-MARCKS and S25p- MARCKS in ganglion cells. Arrowheads, mitotic cells at the apical border of the differentiating retina. gcl, ganglion cell layer; inl, inner nuclear layer; ipl, inner plexiform layer; ofl, optic fiber layer. Scale bars: 20 µm.

To determine if PMA was able to stimulate PKC and promote MARCKS ED phosphorylation in the neural retina, the whole organ was isolated from E8 chick embryos and incubated in the presence of increasing PMA concentrations during 30 minutes, in serum-free medium. The tissues were solubilized and the proteins separated by SDS-PAGE, electrotransferred to nitrocellulose membranes and serially immunodetected with the different anti-MARCKS antibodies. As Figure 6A shows, the antibody against phosphorylated ED recognized a broad electrophoretic band when the cells were treated with 0.3 and 3 µM PMA (lower concentrations only provoked a slight phosphorylation). In the same conditions it was observed that the amount of S25p-MARCKS was barely detected in the treatments with the highest PMA concentration (Fig. 6A). The merged colored images demonstrate that the fraction of MARCKS phosphorylated at serine 25 is very small and, as described in previous sections, it occupies the top of the MARCKS band. At the same time, the pED-positive band is nearly as wide as that corresponding to the total protein. Merging the images of the electrophoretic bands corresponding to pED- and S25p-MARCKS it can be clearly appreciated that they are well separated, not overlapping at any point (Fig. 6A). These results, indicating that both MARCKS phosphorylations did not concur in the same protein molecules, prompted us to explore the cellular localization of these variants in retinas treated with the highest PMA concentration.

S25p-MARCKS was found across the whole neural retina width. It only exhibited slight differences in intensity along the retinal plane, in correlation with the central to peripheral differentiation gradient (as it was already described [28]). pED-MARCKS was nearly completely absent, only detectable in some mitotic cells and in neuroblasts located close to the vitreal surface of the neural retina (Fig. 6B, and better shown at the higher magnification images, Fig. 6 on the right). It is of note to observe that pED-MARCKS mainly localized at the cytoplasm, in contrast with S25p-MARCKS which localized at the cell periphery. After the incubation with PMA there was a noticeable increase in pED-MARCKS immunoreactivity, spanning the whole retinal width (Fig. 6C). At the subcellular level, it appeared as if there was a territorial segregation of both phosphorylated MARCKS variants (Fig. 6C and the lateral higher magnification images). To further analyze the subcellular localization of pED- and S25p-MARCKS, neural retina cells were dissociated and cultured up to a stage in which the cells possessed long neurites.

**Figure 7:**
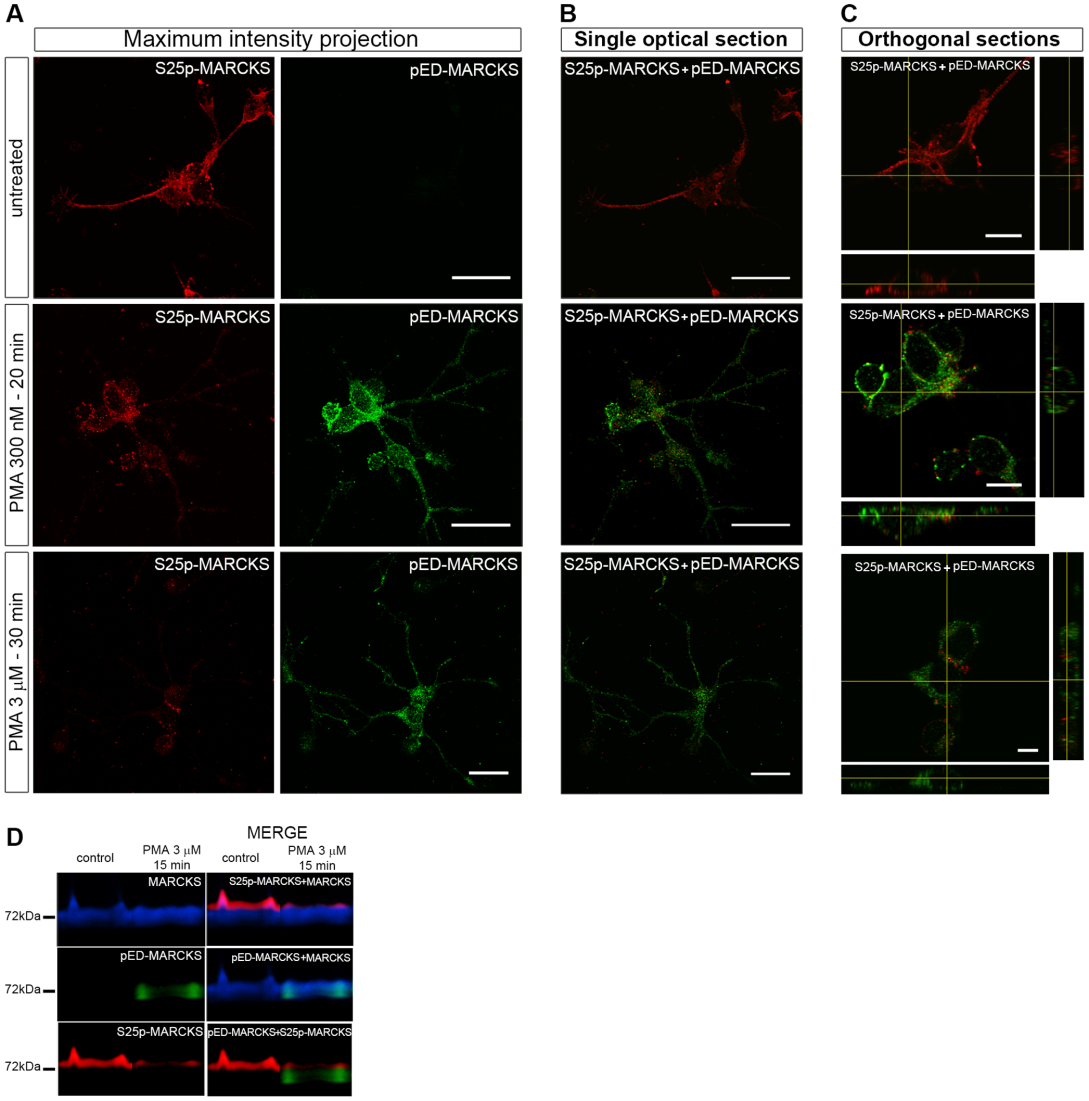
MARCKS phosphorylation at the ED and S25 in E8 neural retina cell cultures stimulated with PMA. (**A–C**) Immunodetections of pED-MARCKS and S25p-MARCKS on 72 hours *in vitro* neural retina cell cultures, treated with different PMA concentrations. The same cells are shown, as maximum intensity projections of the confocal stacks (A) and as merged optical sections at different planes and orthogonal angles (B and C). Scale bars: 10 µm. (**D**) Pseudo-colored sequential Western-blot immunodetections for MARCKS, pED-MARCKS and S25p-MARCKS, of E8 chick embryo neural retina cell cultures.

The videos in supplementary material present 3D reconstructions of E8 chick retina cell cultures untreated (Video S1) or stimulated with PMA for 10 minutes (Video S2). In the untreated cells MARCKS protein is widely distributed in peripheral speckles in neuroblasts as well as in Müller glia cells. On the other hand, S25p-MARCKS is only present in neuroblasts. Almost every MARCKS cluster contains MARCKS phosphorylated at S25. In the treated neuroblast the individual pED- and S25p-MARCKS-containing clusters can be clearly appreciated showing that clusters containing the different phosphorylated isoforms do not co-localize. The intensity of the fluorescent signal arising from the clusters was similar to that observed in cultured retinas after PMA treatment: a noticeable increase of pED-MARCKS and a concomitant decrease of S25p-MARCKS immunoreactivity (Fig. 7A). As an additional evaluation of the co-localization, single optical sections were compared. Figure 7B shows neuroblast sections corresponding to the basal-most cell planes and Figure 7C the orthogonal planes at the central region of one selected cell. As it was also evidenced in these last images there was no co-localization of S25p-MARCKS and pED-MARCKS in the same speckles. These results are consistent with those obtained by Western-blot assays (Fig. 7D).

**Figure 8:**
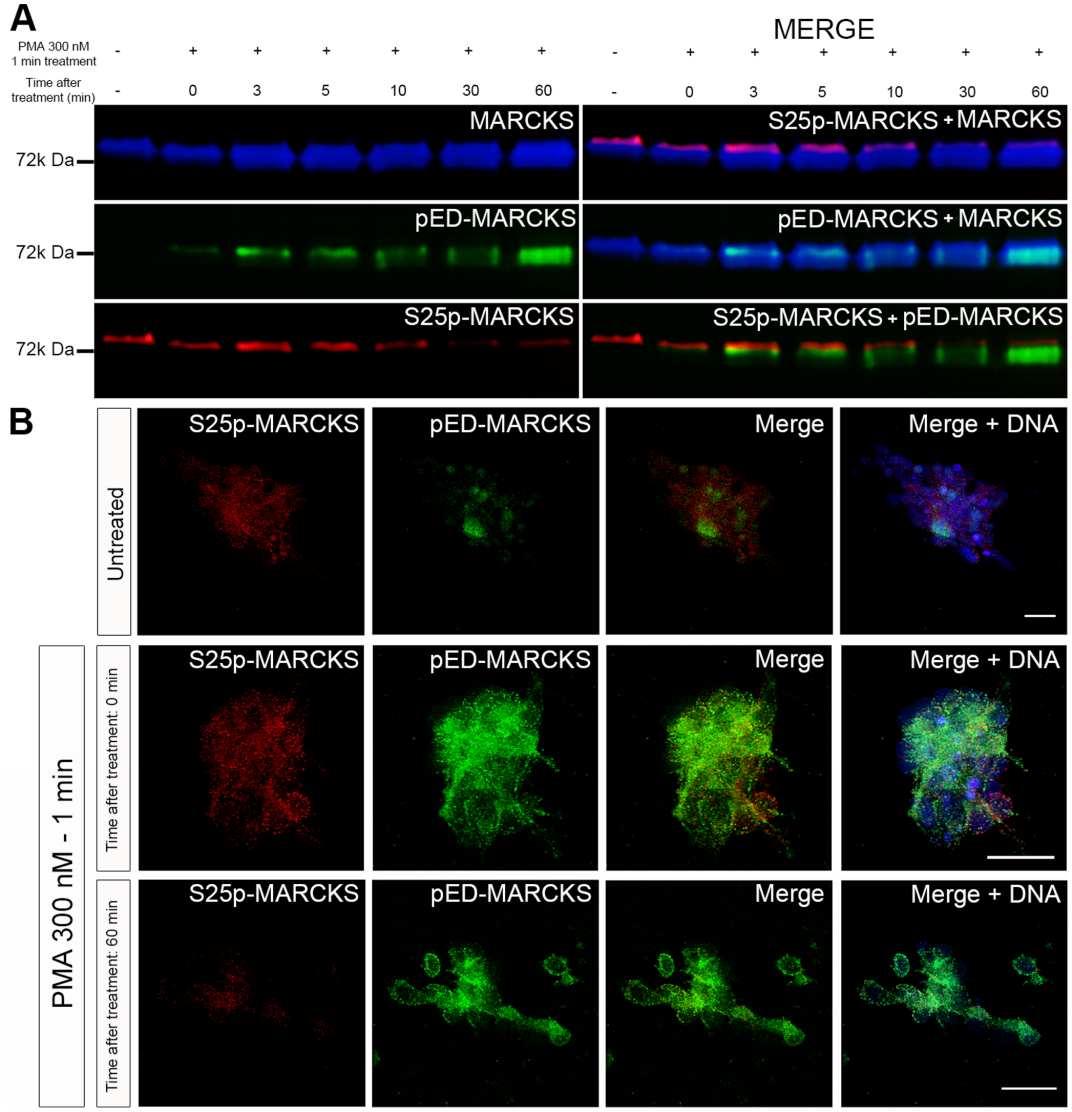
pED-MARCKS persistence in chick retina cell cultures treated with PMA. (**A**) Pseudo-colored sequential Western-blots. E8 chick retina cells were cultured for three days and then treated with 300 nM PMA for one minute, or 0.1% DMSO for the controls. Cultured cells were then washed and kept in fresh culture medium without PMA for different times as detailed. (**B**) Immunodetection of S25p-MARCKS and pED-MARCKS on chick retina cell cultures untreated, treated with 300 nM PMA for one minute and immediately fixed, or treated with 300 nM PMA for one minute and left in fresh medium for 60 minutes. Images are maximum intensity projections. Scale bars: 20 µm.

Is there a rapid decay in pED-MARCKS and a recovery of S25p-MARCKS after the removal of PMA? Neural retina cells were cultured for 3 days in complete culture medium, they were then washed with serum-free medium during 2 min, this medium was withdrawn and substituted by serum-free medium containing 300 nM PMA for 1 min. The cells were solubilized at different times (0 to 60 min) and processed for immunoblotting with different antibodies. pED-MARCKS appeared evident at the end of the PMA pulse and its amount increased as a function of time, while S25p-MARCKS progressively decreased from 5 to 60 minutes (Fig. 8). Altogether these results showed that: a) PMA is a strong PKC stimulator in neural retinaneuroblasts; b) MARCKS phosphorylation at the ED, consequent to this stimulation, is concomitant with a decrease of its phosphorylation at serine 25; c) the clusters containing one or the other phosphorylated forms are exclusive; and d) there is a sustained PKC activity even in the absence of external stimulation with PMA.

### Which are the Phosphatases Involved in MARCKS S25 Dephosphorylation in Relation to ED Phosphorylation?

**Figure 9:**
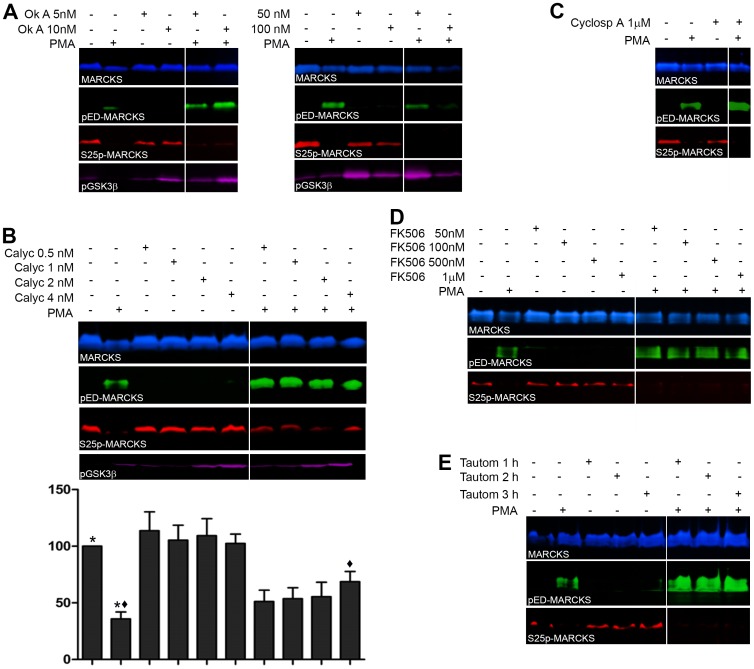
MARCKS phosphorylation at S25 in E8 neural retina cell cultures treated with PMA and phosphatase inhibitors. Sequential western-blot immunodetections for MARCKS, pED-MARCKS, S25p-MARCKS and pGSK-3β of E8 chick embryo neural retina cell cultures treated with PMA and phosphatase inhibitors (okadaic acid, calyculin A, tautomycetin, FK506 and cyclosporin A), in DMEM without serum. Each immunodetection is shown in a different color. Neural retina cell cultures were incubated 72 hours *in vitro*, and then treated with different phosphatase inhibitors or DMSO. After this, PMA 300 nM was added to the cultures for other 10 minutes, without removing the phosphatase inhibitor. (**A**) Cell cultures treated with okadaic acid (5 and 10 nM during 4 hours and 50 and 100 nM during 2 hours) or DMSO. (**B**) Cell cultures treated with calyculin A (0.5, 1, 2 and 4 nM) or DMSO for 15 minutes. Bar diagram represents the relative phosphorylation of MARCKS at S25. The values are represented as mean±SEM of four independent experiments. Statistical significance was determined using the nonparametric tests Kruskal-Wallis and Wilcoxon-Man Whitney; *and ♦ indicate p≤0.05. (**C**) Cultured cells treated with 1 µM cyclosporin A or DMSO for 15 minutes. (**D**) Cell cultures treated with FK506 (50, 100, 500 nM and 1 µM) or DMSO for 15 minutes. (**E**) Cell cultures treated with 1µM tautomycetin or DMSO during 1, 2, and 3 hours.

We explored whether neuroblasts contain a Ser/Thr-protein phosphatase that stimulated by the PMA treatment could account for the observed S25p-MARCKS dephosphorylation. Many years ago it was shown by Yamamoto et al. [11] that purified MARCKS, phosphorylated by proline-directed kinases such as cdc2 (Cdk1) or tau protein kinase II, TPKII (Cdk5), could be dephosphorylated by protein phosphatase-2A (PP2A) and not by calcineurin (PP2B). Very interestingly, it was recently demonstrated that the catalytic activity of PP2A on tyrosine hydroxylase phospho-serine 40 is stimulated by PKC, via the phosphorylation of a regulatory site in the subunit B56d of PP2A [29]. These data constituted the rationale for a search of putative phosphatase(s) involved in the dephosphorylation of MARCKS serine 25, the novel effect of MARCKS phosphorylation by activated PKC described here. The approach chosen was to treat cells with some well-known Ser/Thr phosphatase inhibitors and PMA. PMA was used at the same concentration as in the experiments shown in Figures 7 and 8 (300 nM). The selected inhibitors were okadaic acid (10–300 nM), calyculin A (0.5–8 nM), tautomycetin (100 nM-1 µM), FK506 (50 nM-1 µM) and cyclosporin A (100 nM-1 µM). Cells were pre-treated for variable times with the phosphatase inhibitor, then PMA was added and cells were cultured for 10 min in the presence of both drugs. As Figure 9 shows, untreated cells contain high amounts of S25p-MARCKS, whereas the protein is not phosphorylated at its ED as described above. Once the cells have been treated with PMA during 10 minutes these phosphorylations appeared strictly opposite, with a noticeable decrease in S25 and an increase in ED phosphorylation (Fig. 9A–E). The preincubation with okadaic acid during more than 2 hours was not able to inhibit the dephosphorylation at S25. However, the phosphorylation level of MARCKS ED and GSK3β at its serine 9 appeared increased, indicating that the phosphatase inhibitor was active (Fig. 9A). This figure illustrates the use of four concentrations of this toxin to show how the highest one (100 nM) produced a clear decrease in total protein content due to cell loss. This concentration was found to be the highest usable with neuroblasts, even when applied during shorter times (60 minutes). Higher okadaic acid concentrations provoked massive cell death in the cultures. Given this observation, we suspected than even low concentrations could be inducing apoptosis. We know that there is an early MARCKS S25dephosphorylation when cells undergo apoptosis [30], therefore we used a different Ser/Thr phosphatase inhibitor: calyculin A, which also inhibits PP1. Cells pretreated with calyculin A, at concentrations ranging from 0.5 nM to 8 nM during 25 minutes, exhibited a noticeable attenuation of MARCKS dephosphorylation at serine 25. Figure 9B shows the results corresponding to some of the calyculin A concentrations studied. In the presence of PMA the amount of S25p-MARCKS, evaluated against the total protein transferred and stained with Ponceau Red, decreased to less than 35% of the amount in untreated cells, but when they had been incubated with calyculin A the decrease was only to 70% of the controls (see bar graph in Figure 9B). In addition, the effectiveness of calyculin A as phosphatase inhibitor on these cells was also supported by an evident increase of both pED-MARCKS and pSer9-GSK3β.

The treatment with inhibitors with a higher specificity for PP2B, cyclosporin A and FK506 did not prevent, nor attenuate, MARCKS dephosphorylation at serine 25 (Fig. 9C and D). There was, however, an increase of pED-MARCKS, indicating that its phosphorylation/dephosphorylation balance was displaced towards the phosphorylated state, as an effect of PP2B inhibition. As calyculin, the only drug to have an effect on S25 dephosphorylation upon PMA treatment, inhibits both PP1 and PP2A and each with a not too different IC_50_, we assayed another compound known to be a more specific PP1 inhibitor. Tautomycetin selectively inhibits PP1 over PP2A [31], [32]. Different concentrations and treatment times were used (100 nM-1 µM, from 25 min to 24 h), finding that this inhibitor did not modify the extremely low level of S25p-MARCKS in cells treated with PMA (Fig. 9E). The activity of tautomycetin was clearly evidenced by the concomitant increase of pED-MARCKS. The results presented here also permitted us to obtain information about the sensibility to phosphatases of MARCKS-ED serines 152–156 (phospho-serines recognized by the anti phospho-ED antibody used here). In fact, these results show that all three, PP1, PP2A and PP2B, appear to have the ability to dephosphorylate these serines in retinal neuroblasts (Fig. 9).

Taken together, our results indicate that MARCKS serine 25 is dephosphorylated by PP2A when the cells are stimulated with PMA.

## Discussion

To analyze the relative amounts of two differently phosphorylated MARCKS variants present in the same immunoreactive electrophoretic band, we introduced here a new and simple procedure for processing Western-blot images. Merging digitally colored immunoreactive bands permitted us to obtain a quick semi-quantitative evaluation of polypeptide amounts. This procedure was used to compare the quantity of total MARCKS, pED-MARCKS and S25p-MARCKS during neural retina development, as well as in several different experimental situations. Therefore, it was possible to show that MARCKS was present since the earliest neural retina developmental stage assayed here, and that the same holds for S25p-MARCKS (initially expressed by differentiating retinal ganglion cells), confirming our earlier findings [14]. In addition we showed the presence of pED-MARCKS *in vivo* already at E4, although only the mitotic cells were immunoreactive. It must be remembered that at this developmental stage there is an important cell proliferation activity in the neural retina, which explains why this isoform constitutes a large fraction of the total protein. pED-MARCKS cell distribution has recently been described in dividing human hepatic stellate cells, where MARCKS was highly phosphorylated and localized at distinct sub-cellular regions in each one of the mitotic phases [33]. Developmental changes in the amount of pED-MARCKS have also been found in rat brain, where this MARCKS phosphorylated variant was not detectable in embryos before the 14th day, but increased at birth reaching the highest values at P5–P22 [34]. S25p- and pED-MARCKS displayed very different spatial and temporal expression patterns during neural retina development. While the former accumulated with the progression of cell differentiation and spanned the whole retinal width, the latter appeared to vary randomly and was mainly confined to the retinal ganglion cells and their axons as well as to mitotic cells.

Taking into account the comparison of MARCKS protein sequences from several vertebrate species, a high homology was found in the region named “MH2”, S25 to E49 in the chick sequence (S26-E50 in humans) [35]. Part of this conserved protein domain was initially identified as the one comprising the RNA splicing region, which could be the reason for its high level of preservation [3]. The fact that a phosphorylation site specific for differentiating neurons is highly conserved among all MARCKS sequences identified so far, also suggests that it might be important for its function in those cells. In addition, S25 is inserted in a consensus sequence for Cdk phosphorylation (VAASPSK) recognized by ELM (“Eukaryotic Linear Motif”; [36]) web site as such; and as we mentioned in the Introduction, human MARCKS Serine 26 was phosphorylated by Cdk2 in *in vitro* assays [13].

Our results from kinase inhibition experiments indicate that MARCKS phosphorylation at S25 is maintained by a permanent activity of a Cdk, which must be counteracted in cells by some unidentified phosphatase(s). As cell-cycle related Cdks are not active in differentiating retinal neurons [26], [27], we deduced that the kinase responsible for this modification is Cdk5. Cdk5 is a multiple-target Ser/Thr protein kinase that has been involved in several processes during neuronal differentiation [37], [38], [39]. Many of its known targets are cytoskeleton-modulating proteins, such as Tau and neurofilament proteins, as well as some intracellular signaling proteins. In the case of MARCKS, previous work from Yamamoto and collaborators has shown that Cdk5, as well as Cdk1, are able to phosphorylate some unidentified rat MARCKS serine and threonine residues *in vitro* [11]. However, kinase inhibitors are usually not completely specific, and it has also been shown that there is some extent of interaction of the activity of kinases like Cdk5 and GSK3β, and that they may even phosphorylate some proteins at the same sites [40]. Results from pharmacological experiments shown here demonstrate that GSK3β does not phosphorylate MARCKS at S25 in a detectable way in living cells. Interestingly, the same experiments also indicate that MARCKS phosphorylation by this enzyme would not be necessary for priming the one produced on S25 by Cdk5. This concerted action of both enzymes is a known phenomenon, occurring on Tau, where Cdk5 modulates some GSK3β specific site phosphorylations [41]. The pharmacological inhibition experiments presented here also indicate that Cdk5 would be the site-specific kinase phosphorylating MARCKS, not only at the onset but also at the progression, of retinal neuroblasts differentiation. On the other hand, this same enzyme produces the phosphorylation of MARCKS in cells after a near complete dephosphorylation of S25, when actin filaments are disassembled, as described [16].

Additional information to reinforce the idea that Cdk5 does indeed interact with MARCKS *in vivo*, derives from the observation that both proteins are found accumulated in the same cells in the retina, and partially co-localize in growing neurites and growth cones. Their incomplete subcellular co-localization is most probably related to the fact that, in differentiating neurons, Cdk5 is associated to the cytoskeleton, where it phosphorylates many other substrates [37]. As we found both proteins associated to low-density membrane microdomains (Fig. S2), it is tempting to speculate that these complexes are some type of actin-associated membrane rafts. p35 and p39, Cdk5-activating subunits, are myristoylated proteins [42], hence active Cdk5 could in theory interact at the plasma membrane with the same microdomains as MARCKS. But we cannot rule out other hypothesis: since phosphorylation at S25 appears to be very stable it could also be possible that MARCKS phosphorylation by Cdk5 occurs at a cellular compartment apart to where the protein is actually functional.

We previously showed that the persistence of S25 phosphorylation depends on actin filaments stability [16]. Now we describe that the phosphorylation at MARCKS ED by PKC provokes a rapid S25 dephosphorylation. The attenuation, and even the disappearance of this phosphorylation, occurs as a function of PMA concentration and treatment duration. Moreover, it is enough to stimulate PKC with PMA during one minute to produce two effects: a) a progressive increase of ED phosphorylation, and b) an associated decrease of S25 phosphorylation. Our results concerning ED phosphorylation differ from the early ones obtained by Rozengurt stimulating Swiss 3T3 intact cells with phorbol 12–13 dibutyrate (PBt_2_) for 2 minutes. In these cells, the 80 kDa protein (MARCKS) was rapidly phosphorylated to a maximum followed by a decay attaining minimal values 6 minutes after PBt_2_ withdrawal [43]. As far as we know our results are the first reporting the existence of a relationship between two MARCKS phosphorylated sites, produced by different kinases. It was clear from our results that there is a decay of S25p-MARCKS when Cdk5 is inhibited in intact cells. This decay would be indicating that the involved specific phosphatase is active, but that the phosphorylation is not sustained as a consequence of the kinase inhibition by roscovitine or olomoucin. Interestingly, in conditions of PKC stimulation by PMA, we observed a faster loss of S25 phosphate. This result strongly suggested that PKC would be activating the phosphatase and/or perhaps modifying the kinetics of Cdk5.

It is known from the results of Yamamoto [11], that PP2A is the phosphatase removing the MARCKS phosphates incorporated by proline-directed kinases, like Cdks. An analogous phenomenon was found at the dopamine regulation of DARPP-32 phosphorylation, which occurs at its threonine 75 in neostriatal neurons. In this case it is the cAMP dependent protein kinase (PKA) that, once stimulated by dopamine, increases PP2A activity without modifying the activity of Cdk5; at the same time it phosphorylates DARPP-32 at another residue, threonine 34 [44]. More recently it was reported that the activation of PP2A is due to a phosphorylation of its B56d subunit, produced by PKA [45]. Later on, these authors found that stimulated PKC is able to phosphorylate B56d subunit, leading to an activation of PP2A, which produced the dephosphorylation of Ser 40 of tyrosine hydroxylase [29].

Most of the previous research regarding MARCKS serine dephosphorylation performed either using the purified protein or on different cell types, has been devoted to those residues located at the ED and phosphorylated by PKC. ED dephosphorylation has been attributed to phosphatases PP1A, PP2A, PP2B and PP2C [46], [47], [48], [49], [50], [51]. Nevertheless, MARCKS has several other serine and threonine residues known to be phosphorylated by different protein kinases (see review by Mosevitsky [5]), although only a few experiments explored their dephosphorylation *in vitro*. In fact, Yamamoto et al. [11] showed that MARCKS, phosphorylated by PKC, cdc2 or Cdk5, was only dephosphorylated by the holoenzyme of PP2A and not by PP2B. We assayed the effect of various protein phosphatase inhibitors in several treatments applied to intact neuroblasts. The results presented here show that only calyculin A attenuates the dephosphorylation induced by PMA treatment. All the drugs had clear inhibitory effects over phosphatases acting on the phosphorylated MARCKS ED or, in the case of okadaic acid and calyculin A, on GSK3β phospho-serine 9, indicating that in neuroblasts both substrates are sensitive to phosphatase inhibition with okadaic acid. It is of note that in other cell types such as Swiss 3T3, okadaic acid inhibits phosphatase 2A acting on MARCKS ED at concentrations that are extremely toxic for neuroblasts [47].

From our experiments, PP1 and PP2B can be excluded as putative phosphatases for MARCKS serine 25, given the non-inhibitory effects of tautomycetin (PP1), FK506 and cyclosporine A (PP2B). Experiments using calyculin A as protein phosphatase inhibitor pointed to a form of PP2A as the most probable phosphatase involved in MARCKS S25 dephosphorylation. It is known that this enzyme is highly versatile depending of its regulatory subunit (reviewed in [52]). It has been demonstrated that PKC phosphorylation of a PP2A subunit causes an increase of its catalytic activity, as described by Ahn et al. [29] for tyrosine hydroxylase dephosphorylation. This would be considered a potential explanatory mechanism for MARCKS dephosphorylation under our experimental conditions.

The cellular phenomenon described here appears singularly complex, particularly in which concerns to the molecular mechanisms that would involve structural aspects as well as regulatory networks. In fact, it is necessary to elucidate the dynamic structural properties of the MARCKS region in which S25 lies, and that could account for the peculiarities of its phosphorylation/dephosphorylation cycle in cells. At the same time it is necessary to gain a better understanding of many intricate signal transduction networks acting during neuroblast differentiation.

## Supporting Information

Figure S1 **Characterization of anti-MARCKS antibody (Polo52).** Western-blot of E12 chick neural retina. A 0.2% Triton X-100 retina lysate containing 10 µg of protein was separated in 10% SDS-PAGE and sequentially immunodetected with anti-MARCKS polyclonal antibodies (Polo52, MCt and Martin) and mAb 3C3. Observe that Polo52 antibody recognizes only one broad band at around 70 kDa. It behaves as the other anti-MARCKS antibodies MCt and Martin D202-D87.
(TIF)

Figure S2 **Dissection and culture of differentiating neuroblasts from peripheral E4.5 chick retinas.** Neuroephitelial cells were isolated form peripheral regions of E4.5 chick retinas. These dissociated cells differentiate in culture as retinal ganglion cells (RGCs) expressing the specific marker RA4 [53], closely followed by the phosphorylation of MARCKS at S25. This provided us with a culture system in which most of the cell population was actively accumulating S25p-MARCKS from a totally unphosphorylated state. **(A)** Schematic representation of an extended E4.5 chick neural retina, showing the dissection procedure followed to obtain the retinal neuroepithelial cells. ONH, optic nerve head. **(B)** Percent of S25p-MARCKS- and RA4-positive cells as a function of time, in cultures from E4.5 peripheral neural retina cells. The dotted line represents the percent of cells with fragmented nuclei (in advanced stages of apoptosis). S25p-MARCKS and RA4 values were normalized to mean values at 2 hours after seeding. Values are represented as mean±SEM, n = 2 independent cultures, counting 1000–2000 cells in each. The ascending RA4 and S25p-MARCKS graphs show that at 24 hours most of the neurons differentiated *in vitro*. **(C)** General aspect of neuroepithelial cell cultures 24 hours after explantation. **(D)** Cultured neuroblasts exhibiting growing neurites 24 hours after explantation. Scale bars: C, 50 µm; D, 7 µm.
(TIF)

Figure S3 **Flotation assays.** Sequential Western-blots showing the presence of total MARCKS, S25p-MARCKS and Cdk5 in low-density membranes (rafts) obtained after sucrose-gradient centrifugation of a lysate from chick embryo brains (E12). The raft fraction is characterized by the enrichment of the 120 kDa N-CAM isoform.
(TIF)

Table S1 **Assessment of cell death/suffering signs after a prolonged treatment of E4 peripheral retina cell cultures with the Cdk inhibitor roscovitine.**
(PDF)

Video S1 **3D reconstruction of untreated E8 chick retina cell cultures. MARCKS is labeled in green and S25p-MARCKS in red.** Note the presence of MARCKS protein in neuroblasts and Müller glia, while S25p-MARCKS is only present in neuroblasts. Both, MARCKS and S25p-MARCKS are localized in peripheral speckles in neuroblasts and almost every MARCKS cluster contains phosphorylation at S25.
(MP4)

Video S2 **3D reconstruction of E8 chick retina cell cultures treated with 300 nM PMA during 10 minutes.** S25p-MARCKS is shown in red and pED-MARCKS in green. Note the reduction of S25p-MARCKS compared to the untreated situation (Video S1). Also, pED- and S25p-MARCKS barely co-localize at the cluster level.
(MP4)
