# Supplementary figures and images for: A Novel Effect of MARCKS Phosphorylation by Activated PKC: The Dephosphorylation of Its Serine 25 in Chick Neuroblasts
Source: PLoS One. 2013 Apr 25;8(4):e62863. doi: 10.1371/journal.pone.0062863 (PMC3636281; doi:10.1371/journal.pone.0062863)

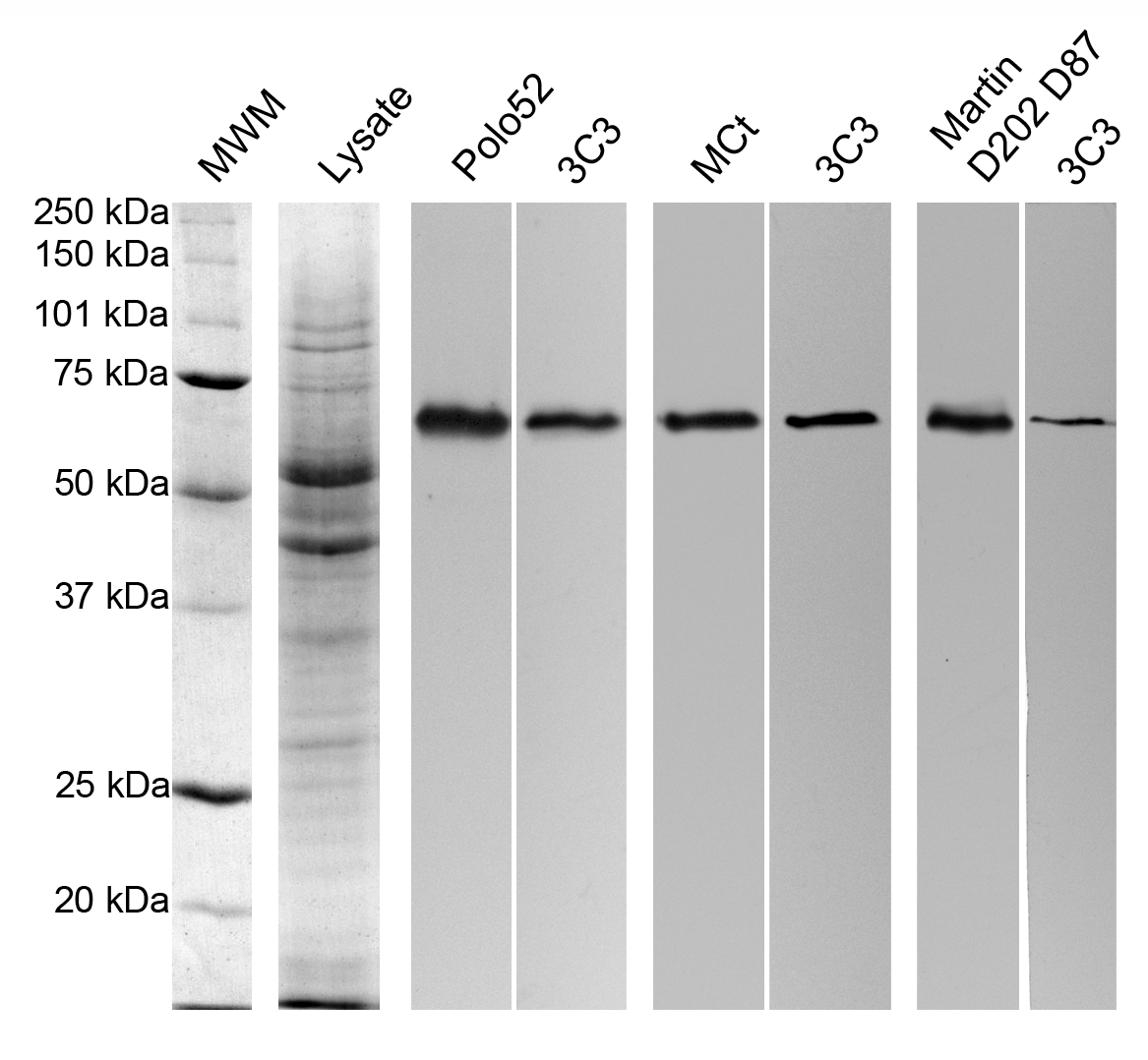

Supplement: Figure S1 — Characterization of anti-MARCKS antibody (Polo52). Western-blot of E12 chick neural retina. A 0.2% Triton X-100 retina lysate containing 10 µg of protein was separated in 10% SDS-PAGE and sequentially immunodetected with anti-MARCKS polyclonal antibodies (Polo52, MCt and Martin) and mAb 3C3. Observe that Polo52 antibody recognizes only one broad band at around 70 kDa. It behaves as the other anti-MARCKS antibodies MCt and Martin D202-D87. (TIF) [file pone.0062863.s001.tif]

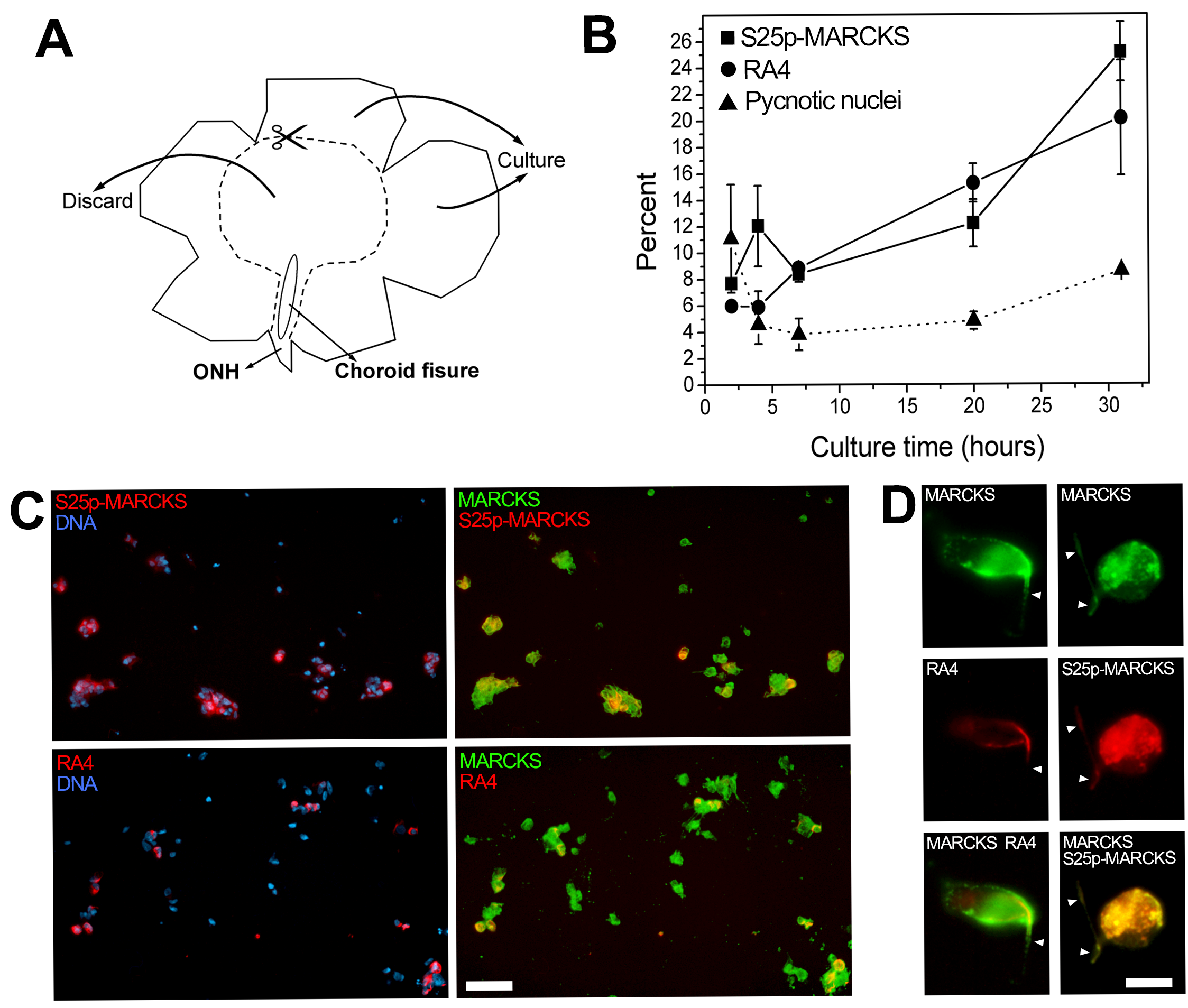

Supplement: Figure S2 — Dissection and culture of differentiating neuroblasts from peripheral E4.5 chick retinas. Neuroephitelial cells were isolated form peripheral regions of E4.5 chick retinas. These dissociated cells differentiate in culture as retinal ganglion cells (RGCs) expressing the specific marker RA4 [53], closely followed by the phosphorylation of MARCKS at S25. This provided us with a culture system in which most of the cell population was actively accumulating S25p-MARCKS from a totally unphosphorylated state. (A) Schematic representation of an extended E4.5 chick neural retina, showing the dissection procedure followed to obtain the retinal neuroepithelial cells. ONH, optic nerve head. (B) Percent of S25p-MARCKS- and RA4-positive cells as a function of time, in cultures from E4.5 peripheral neural retina cells. The dotted line represents the percent of cells with fragmented nuclei (in advanced stages of apoptosis). S25p-MARCKS and RA4 values were normalized to mean values at 2 hours after seeding. Values are represented as mean±SEM, n = 2 independent cultures, counting 1000–2000 cells in each. The ascending RA4 and S25p-MARCKS graphs show that at 24 hours most of the neurons differentiated in vitro. (C) General aspect of neuroepithelial cell cultures 24 hours after explantation. (D) Cultured neuroblasts exhibiting growing neurites 24 hours after explantation. Scale bars: C, 50 µm; D, 7 µm. (TIF) [file pone.0062863.s002.tif]

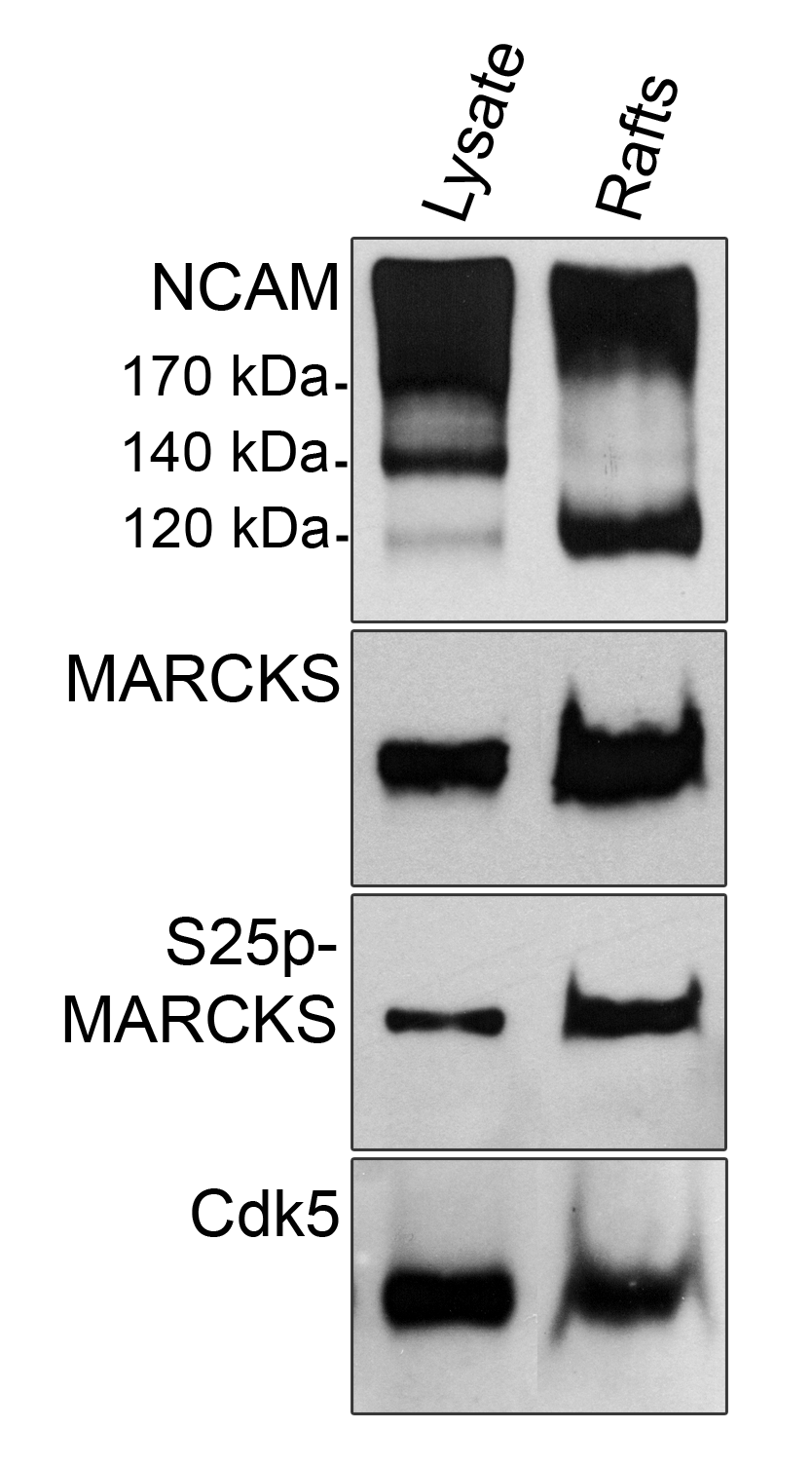

Supplement: Figure S3 — Flotation assays. Sequential Western-blots showing the presence of total MARCKS, S25p-MARCKS and Cdk5 in low-density membranes (rafts) obtained after sucrose-gradient centrifugation of a lysate from chick embryo brains (E12). The raft fraction is characterized by the enrichment of the 120 kDa N-CAM isoform. (TIF) [file pone.0062863.s003.tif]
