# Supplementary material for: A Novel Effect of MARCKS Phosphorylation by Activated PKC: The Dephosphorylation of Its Serine 25 in Chick Neuroblasts
Source: PLoS One. 2013 Apr 25;8(4):e62863. doi: 10.1371/journal.pone.0062863 (PMC3636281; doi:10.1371/journal.pone.0062863)
Supplement: Table S1 — Assessment of cell death/suffering signs after a prolonged treatment of E4 peripheral retina cell cultures with the Cdk inhibitor roscovitine. (PDF) [file pone.0062863.s004.pdf]

Supplementary Table 1. Assessment of cell death/suffering signs after a prolonged treatment of E4 peripheral retina cell cultures with the Cdk inhibitor roscovitine.

	**Percent of cells with pyknotic nuclei**	**Percent of cells with surface blebbing**
**DMSO, 5h**	12.4 ± 1.5	7.2 ± 0.8
**25 μM Roscovitine, 5h**	14.2 ± 0.6	6.4 ± 0.7

Mean ± SE, N=3 different cultures, counting 1000-2000 cells/culture. Nuclei were stained with Hoechst 33342, and cell surface with anti-total MARCKS antibody (“MCt”).
